# Injection of Testicle Juice in Tuberculosis

**Published:** 1892-10

**Authors:** 


INJECTIONS OF TESTICLE JUICE IN TUBERCULOSIS.—Espagne and Pourquier (*Nouv. Montpellier Med.*, June 4, 1892) have tried hypodermic injection of testicle juice in a case of pulmonary tuberculosis. The patient was a girl, aged eighteen, without known hereditary antecedents, but of lymphatic temperament. There was harsh breathing nearly all over the chest, and dry crackling at both apices, especially on the left side and at the back. The girl suffered from amenorrhœa and profuse night-sweats, and was wasting steadily. The testicle juice was prepared as follows: 50 grams of testical substance (from a bull calf) were macerated for twenty-four hours in 50 grams of sterilized glycerine. This preparation, after filtration first through paper and then through a Chamberland filter, gave a clear liquid almost as transparent as distilled water. The injection of a Pravaz syringeful of this liquid caused considerable pain, but was followed by a fall of temperature and reduction in the pulse-rate. The authors, however, are doubtful whether a repetition of the injection will be permitted.—*British Medical Journal.*

